# Oxidative Stress in Chagas Disease

**DOI:** 10.1155/2009/190354

**Published:** 2009-06-14

**Authors:** Shivali Gupta, Jian-Jun Wen, Nisha Jain Garg

**Affiliations:** ^1^Department of Microbiology and Immunology, University of Texas Medical Branch, Galveston, TX 77555, USA; ^2^Department of Pathology, University of Texas Medical Branch, Galveston, TX 77555, USA; ^3^Sealy Center for Vaccine Development, Institute for Human Infections and Immunity, University of Texas Medical Branch, Galveston, TX 77555, USA; ^4^3.142C Medical Research Building, University of Texas Medical Branch, 301 University Boulevard, Galveston, TX 77555-1070, USA

## Abstract

There is growing evidence to suggest that chagasic myocardia are exposed to sustained oxidative stress induced injuries that may contribute to disease progression. Trypanosoma cruzi invasion- and replication-mediated cellular injuries and immune-mediated cytotoxic reactions are the common source of reactive oxygen species (ROS) during acute infection. Mitochondria are proposed to be the major source of ROS in chronic chagasic hearts. However, it has not been established yet, whether mitochondrial dysfunction is a causative factor in chagasic cardiomyopathy or a consequence of other pathological events. A better understanding of oxidative stress in relation to cardiac tissue damage would be useful in the evaluation of its true role in the pathogenesis of Chagas disease and other heart diseases. In this review, we discuss the evidence for increased oxidative stress in chagasic disease, with emphasis on mitochondrial abnormalities, and its role in sustaining oxidative stress in myocardium.

## 1. Chagas Disease

Chagas disease continues to pose a serious threat to health in Latin America and Mexico, and is the most important emerging parasitic disease in developed countries. According to the World Health Organization, the overall prevalence of human *Trypanosoma cruzi* infection is at ~16–18 million cases, and ~120 million people are at risk of infection in Latin America [1]. In most patients, the early period of *T. cruzi * infection goes virtually unnoticed whereas others develop an acute phase that lasts several weeks and is accompanied by such nonspecific symptoms, fever, tachycardia, weakness, and lymphadenopathy [2, 3]. After acute control of *T. cruzi*, infected patients enter an indeterminate phase, defined by the absence of clinical symptoms although subclinical pathology may be present. Unfortunately, 15–30 years after the initial infection, 30–40% of the infected patients develop life threatening dilated cardiomyopathy associated with clinical symptoms of ventricular dilation, arrhythmia, and cardiac arrest [4]. The pathological developments and clinical symptoms vary widely among chagasic patients [2, 5–7]. Not every individual infected with *T. cruzi * experiences the abnormalities characteristic of the three phases of Chagas disease: acute, indeterminate, and chronic. These facts make Chagas disease a complex disease and difficult to understand.

Over the years, a number of mechanisms have been proposed to explain the pathogenesis of Chagas disease (reviewed in [8, 9]). There is growing evidence to suggest that chagasic myocardia are exposed to sustained oxidative stress-induced injuries that may contribute to disease progression. In this review, we discuss the evidence for increased oxidative stress in chagasic disease, with emphasis on mitochondrial abnormalities, as well as electron transport chain dysfunction, and its role in sustaining oxidative stress in myocardium.

## 2. Sources of Oxidants

### 2.1. Overview

Broadly defined, reactive oxygen species (ROS, e.g., O_2_^• −^, ^•^OH, and H_2_O_2_) are derivatives of molecular oxygen. ROS are unstable and react rapidly with other free radicals and macromolecules in chain reactions to generate increasingly harmful oxidants. ROS are produced through the action of specific oxidases and oxygenases (e.g., xanthine oxidase, and NADPH oxidase), peroxidases (e.g., myeloperoxidase), the Fenton reaction, and are also by-products of the electron transport chain of mitochondria [10]. Nitric oxide (^•^NO) is produced by the enzymatic activity of nitric oxide synthases (NOS), which oxidize L-arginine, transferring electrons from NADPH. Different NOS isoforms have been identified, for example, inducible NOS (iNOS) in phagocytic cells, mtNOS in mitochondria, (eNOS) in endothelial cells, and neuronal nNOS [11].

### 2.2. ROS in Chagasic Hosts

During the course of *T. cruzi * infection and disease development, ROS can be produced as a consequence of tissue destruction caused by toxic secretions of parasite, immune-mediated cytotoxic reactions, and secondary damage to mitochondria. 

In experimental studies, *T. cruzi * infection has been suggested to initiate ROS formation via the stimulation of inflammatory mediators, for example, cytokines and chemokines, which lead to an oxidative burst of phagocytic cells. Several investigators have used in vitro assay systems or animal models and demonstrated that *T. cruzi*-mediated macrophage activation results in increased levels of O_2_^•−^ formation, likely by the NADPH oxidase-dependent oxidative burst [12–14]. In addition to ROS, activated macrophages can produce large amounts of ^•^NO by iNOS. Accordingly, TNF-*α*- and IFN-*γ*-dependent increased iNOS expression and ^•^NO production is noted in splenocytes of *T. cruzi*-infected mice [15] and in macrophages infected in vitro with *T. cruzi * [16]. We have found increased levels of myeloperoxidase and nitrite in the plasma of *T. cruzi*-infected mice [17] that are markers of neutrophil and macrophage activation, respectively. Relatively few studies have been performed to elucidate inflammatory oxidative stress in human patients. In humans, the severity of cardiac disease was correlated with high plasma levels of TNF-*α* and ^•^NO [18]. The ^•^NO level was also increased in indeterminate individuals in comparison to healthy controls [19]. These reactive oxidants are important for the control of *T. cruzi*, and may elicit toxicity to host cellular components. 

Recent studies provide evidence for enhanced mitochondrial ROS generation (H_2_O_2_ and O_2_^•−^) in chagasic myocardium. Mitochondria are the prime source of energy and many of the body's functions, including those of cardiac metabolic and contractile activities, require mitochondrial generation of ATP. Electron microscopic analysis of heart biopsies from chagasic patients and experimental animals have shown that with disease development, mitochondrial degenerative changes, that is, swelling, irregular membranes, and loss of cristae, accrue in the heart with disease development [20–23]. Global microarray profiling of gene expression has identified alterations in several of the mitochondrial function related transcripts in the myocardium of infected humans [24] and experimental animals [25, 26]. The biochemical evidence for the mitochondrial dysfunction was provided by documentation of a decline in the activities of respiratory complexes, NADH-ubiquinone reductase (CI) and ubiquinol-cytochrome c reductase (CIII) [27] and ATP synthase (CV) complex [28] in chagasic murine hearts. The functional effect of these perturbations was shown by decreased mitochondrial respiration [29], and reduction in myocardial and mitochondrial ATP levels [30] in chagasic experimental models. 

Imperatively, mitochondrial dysfunction also contributes to increased oxidative stress. A low, but constant, production of superoxide O_2_^•−^ occurs in mitochondria. The rate of electron leakage and O_2_^•−^ formation in mitochondria is closely related to the coupling efficiency between the respiratory chain and oxidative phosphorylation [31]. The CI and CIII complexes are the main sites for electron leakage to O_2_ and O_2_^•−^ generation in mitochondria [32, 33]. We have shown a decline in complex I and complex III activities in the myocardium was associated with excessive leakage of electrons to molecular oxygen and sustained ROS production in chagasic mice [27]. Further studies identified that CI was not the main source of increased ROS in chagasic hearts. Instead, defects of the myxothiazol-binding site in CIII complex resulted in enhanced electron leakage towards the Q_o_-center, and contributed to increased ROS generation in chagasic cardiac mitochondria [34]. Thus, conditions conducive to oxidative stress are presented in the Chagasic heart.

## 3. Antioxidants

### 3.1. Overview

The overall level of cellular ROS and its biological effects are determined by the relative rates of ROS generation and the rate of reduction by antioxidants. The principal enzymatic antioxidants are superoxide dismutase (SOD), catalase (CAT), peroxiredoxin (Prx), and glutathione peroxidase (GPx). These enzymes work in tandem to scavenge ROS. SOD exists in different isoforms, for example, manganese SOD (MnSOD) in the mitochondrial matrix and Cu- or Zn-SOD in the cytoplasm, mitochondria intermembrane space, and endothelial cell surface [35]. SOD converts O_2_^•−^ to H_2_O_2_ [36]. CAT, located in peroxisomes, converts H_2_O_2_ to H_2_O and O_2_ [37]. Prx reduces peroxides, including H_2_O_2_ and alkyl hydroperoxides [38]. The five isoforms of GPx utilize glutathione (GSH), and reduce H_2_O_2_ or lipid peroxides (ROOH) to H_2_O or alcohols (ROH), respectively. The byproduct of this reaction, GSSG is recycled by glutathione S reductase [38]. The nonenzymatic antioxidants, for example, vitamin E (*α*-tocopherol) and vitamin C (ascorbate), are abundant in aerobic organisms. Vitamin E, active in membranes, functions to reduce peroxy radicals. Vitamin C, a highly soluble antioxidant in plasma, functions by reducing *α*-tocopherol-lipid peroxide radicals, particularly formed in reaction with the low-density lipoproteins (LDL) [37].

### 3.2. Antioxidant Status in Chagasic Host

The myocardium contains high concentrations of various nonenzymatic antioxidants such as reduced glutathione (GSH) and vitamins A, C, and E, and enzymatic scavengers of ROS, including GPx and Mn- and CuZn-SOD. GSH, GPx, and MnSOD are shown to be most critical in cardiac antioxidant defenses, particularly in protecting the cardiomyocytes from oxidative injury [39, 40]. We and others have evaluated the antioxidant/oxidant balance in experimental models of chagasic disease and human patients. Our experimental studies showed that the host responds to acute *T. cruzi * infection by upregulating glutathione antioxidant defense constituted by GPx, GSR, and GSH. However, after the initial burst, the glutathione defense was unresponsive to chronic oxidative stress, and the cardiac levels of GSH and MnSOD were significantly diminished in chagasic mice [41]. A decline in plasma levels of GSH, the GSH/GSSG ratio [42, 43], and GPx activity [18], along with decreased MnSOD activity in PBMCs of seropositive chagasic patients [42, 43] is also noted. Decreased antioxidant levels (GPx and SOD) were correlated with an increase in TNF-*α* and ^•^NO levels in human patients [18]. All of these observations suggest an antioxidant response is not sufficiently activated to scavenge the ROS during progressive chagasic disease.

## 4. Cytotoxicity of Oxidative Stress

### 4.1. Overview

ROS and ^•^NO, when produced in physiological quantities, play critical roles in normal developmental processes, and control signal transduction mechanisms that regulate cell proliferation, differentiation, and death [44, 45]. However, when ROS are produced in excess or for sustained periods, they may exert toxic effects that damage cells and tissues, thereby resulting in dysfunction of physiological processes. ROS can rapidly oxidize proteins, lipids, and DNA. Lipid peroxidation causes damage to membrane integrity and loss of membrane protein function. Specifically, 4-hydroxynonenal (HNE) and malonyldialdehyde (MDA) are products of the peroxidation of membrane phospholipids [46–48]. These oxidized lipids are also toxic because they are highly reactive species that result in oxidative modification of proteins [37]. For example, HNE reacts with Cys, His, or Lys residues via a Michael addition that results in irreversible alkylation and introduction of carbonyl groups into proteins [49]. The direct oxidative attack by ROS on Arg, Lys, Pro, and Thr residues can also derivatize the proteins and lead to the formation of protein carbonyls [50, 51]. ^•^NO reacts with O_2_^•−^, to form peroxynitrite (ONOO^–^). Myeloperoxidase-dependent oxidation of nitrite (NO_2_^−^) results in formation of nitrogen dioxide (NO_2_) and nitryl chloride (NO_2_Cl). These reactive nitrogen species (RNS) result in protein tyrosine nitration that is widely recognized as a hallmark of nitrosative stress and inflammation [52]. Because of oxidation or nitration, a functional impairment of proteins occurs, and furthermore leads to protein turnover, for example, degradation by proteases via the proteosome [53]. DNA can be oxidized by a variety of mechanisms, resulting in nucleotide damage, for example, formation of 8-oxoguanine lesions. As a result, DNA replication may be inaccurate leading to mutations and transcription errors. While mechanisms exist to repair these DNA lesions, the level of DNA damage may exceed the capacity of the cellular repair mechanisms. Furthermore, mtDNA is believed to be particularly susceptible to sustained damage, since mitochondria may lack appropriate DNA repair mechanisms [54].

### 4.2. Oxidative Damage in Chagas Disease

Oxidative stress-induced injuries are a common finding in chagasic myocardium. *T. cruzi * has the potential to infect a wide range of host tissues [55]. As discussed above, the inflammatory infiltrate in acutely infected host is mainly constituted of phagocytic cells (e.g., macrophages) and neutrophils that produce ROS/RNS through oxidative burst [56], iNOS-dependent ^•^NO release [15], and myeloperoxidase-dependent HOCl production [57]. Oxidative damage is a consequence of the extent of oxidative stress and the antioxidant capacity. A *T. cruzi*-infected host does respond to inflammatory oxidative stress by an upregulation of antioxidant response constituted of GPx, GSH, and GST [41]. Yet, oxidative cellular damage, evidenced by increased protein carbonyls, MDA, and GSSG levels, is widespread, and associated with the presence of parasite foci and inflammatory infiltrate in the heart, as well as in other muscle tissues in acutely infected mice [58]. The acute oxidative damage, thus, appears to be a bystander effect of inflammatory responses elicited by *T. cruzi,* and occurs in all muscle tissues. 

The immune control of acute parasitemia fails to provide sterile immunity. The evolution of a chronic phase is associated with mild-to-moderate diffused inflammation in different tissues and organs. It would be an oversimplification to suggest that cardiac pathology is merely an outcome of infection and inflammation, or parasite persistence that is sufficient to drive an ongoing host immune response targeted against *T. cruzi*. An unvarying high degree of oxidative damage persists mainly in the myocardium of chronically infected mice, as evidenced by high levels of MDA, protein carbonyl, and GSSG contents in the heart compared to findings in the skeletal muscle and colon tissue [58]. We propose the persistent activation of oxidative injurious processes plays an important role in heart-specific tissue damage in Chagas disease. 

Several observations led us to consider that ROS in chronic chagasic heart are primarily produced by dysfunctional mitochondria. It is well known that ROS are generated at several subcellular sites [59] and particularly in mitochondria [60]. In effect, ~2% of the O_2_ consumed by mitochondria is converted to O_2_^•−^ due to spontaneous electron leaks from the respiratory chain [61]. Activated skeletal and intestinal muscles intermittently require mitochondria as an energy source, while cardiomyocytes are constantly dependent upon mitochondrial functions for their energy requirement for maintaining the contractile and other metabolic activities. According to energy demand, a ~30% cell volume of cardiomyocytes is provided by mitochondria, while in other tissues mitochondria constitute only 3–6% of cell volume [62]. Thus, maximal O_2_ consumption, as would be expected based upon the number of mitochondria in the heart, would produce substantial O_2_^•−^ in the heart through electron leakage from the respiratory chain. Thus, it can be inferred that even in normal conditions, heart tissue is maximally exposed to ROS of mitochondrial origin. Besides this, inefficient functioning of the respiratory complexes, as documented in chagasic hearts [27], would result in an inadequate coupling of the respiratory chain with oxidative phosphorylation and an excessive release of electrons to molecular oxygen, leading to an increased mitochondrial ROS production. We have recently found that the rate of mitochondrial O_2_^•−^ generation was substantially increased in cardiac tissue of infected mice [34], and associated with the oxidation of several subunits of the respiratory complexes [41]. The active-site thiol and heme proteins within respiratory complexes are particularly vulnerable to ROS [63]. The oxidative modification/degradation of heme proteins of the complexes release iron, the catalyst of the Fenton reaction, resulting in the formation/release of ^•^OH radicals [64–66]. Taken together, these observations suggest that, under disease conditions, mitochondria are vulnerable to oxidative stress, as well as to becoming the site of an increasing order of ROS production. We, thus, propose that the acute inflammatory oxidative stress-induced mitochondrial injuries initiate a feedback cycle of ROS production and oxidative overload that causes sustained oxidative damage in the myocardium. A compromise in mitochondrial antioxidant enzyme activity (MnSOD) in chagasic myocardium would further exacerbate the mitochondrial ROS toxicity. The foregoing studies have pointed to the pathologic significance of oxidative responses in Chagasic cardiomyopathy.

It is important to note that a high degree of oxidative stress is detected in the peripheral blood of chagasic mice [58]. The demonstration of a strong positive correlation in the heart-versus-blood levels of oxidative stress markers (MDA and GSSG), and antioxidants (SOD, MnSOD, and catalase), and the mitochondrial inhibition of respiratory complexes in chronically infected mice have made it apparent that peripheral blood will be useful for understanding the role of mitochondrial decay and oxidative stress in the initiation and progression of human chagasic disease. 

Subsequently, observations of increased plasma levels of GSSG and MDA and a decline in GPx activity in seropositive humans [18, 42] have led to the suggestion that chagasic patients are indeed exposed to an antioxidant/oxidant imbalance. As in experimental studies, multiple mechanisms are likely to contribute to increased oxidative stress-induced damage in chagasic patients. Plasma levels of inflammatory cytokines, ^•^NO [18] and myeloperoxidase activity [17] are increased in seropositive subjects which seems to imply that the cytotoxic effects of free radicals released by immune cells would contribute to oxidative pathology in chagasic patients. The increase in plasma MDA levels in chagasic patients may also be due to oxidatively modified lipids released as a consequence of cellular injuries, most likely, that are incurred in the cardiac tissue. This notion is supported by the observation of intense myocardial oxidative modifications [41] associated with the detection of oxidatively modified lipids and proteins in the serum [58] of mice infected by *T. cruzi.* Additionally, SOD and glutathione (GPx-GSH-GR) antioxidant defenses, utilized by mammalian cells to cope with free radicals [67], are found to be compromised in chagasic patients [18, 42]. These observations support the idea that glutathione antioxidant defenses, despite being active, may only be partially effective in balancing the oxidant level in chagasic patients.

## 5. Antioxidant Adjunct Therapy

Interventions that reduce the generation or the effects of ROS may exert beneficial effects in preventing or arresting oxidative damage. Several therapeutic interventions, for example, a vitamin E-like antioxidant, an SOD mimetic [68, 69], and an ONOO^–^ decomposition catalyst [70] have been examined for their beneficial effects against ROS in different systems. Phenyl-*N*-*tert*-butylnitrone (PBN), a nitrone-based compound, is a potent antioxidant. PBN has been shown to trap or scavenge a wide variety of free radical species, including biologically relevant O_2_^•−^ and hydroxyl ^•^OH radicals; to increase endogenous antioxidant levels; and to inhibit free radical generation [71]. In addition, PBN has been shown to inhibit the expression of a variety of inflammation-associated gene products [72]. 

In a recent study, we have shown that PBN treatment of infected mice prevented an oxidative stress-mediated loss in mitochondrial membrane integrity; preserved redox potential coupled with mitochondrial gene expression, and improved respiratory complex activities in infected myocardium [30]. Importantly, the PBN-mediated normalization of respiratory complex activities led to the inhibition of a feedback cycle of electron transport chain inefficiency, increased ROS production, and energy homeostasis in acute chagasic hearts [30]. Others have shown a decline in oxidative stress in human chagasic patients given Vitamin A [73]. We propose that antioxidants capable of modulating or delaying the onset of oxidative insult and mitochondrial deficiencies in the myocardium would prove to be useful in preserving cardiac functions in Chagas disease.

## 6. Ischemic Injury and ROS

Approximately 10% of chronic chagasic patients exhibit signs of ischemic disease [74, 75]. The abnormalities during isovolemic contraction and the early relaxation phase, in general ascribed to asynchronous onset of contraction, are noted in chagasic patients, and are similar to that seen in patients with conventional ischemic heart disease of other etiologies [76]. Others have suggested the alterations in the coronary microcirculation contribute to ischemic tissue damage in chronic chagasic patients [75, 77–80]. Myocardial hypoperfusion owing to an affected microvasculature has also been noted in chagasic heart regions with normal or mildly impaired wall motion [75, 80]. 

Hypoxia is a critical outcome of ischemia. In hypoxic tissues, low availability of oxygen results in electron accumulation in highly reduced respiratory complexes that lead to severely compromised respiration and ATP synthesis [81–83]. Ischemia also influences mitochondrial function via change in calcium flux [84], cyt c depletion (reviewed in [85]), and decline in intrinsic level of MnSOD—the mtROS scavenger [86]. The inefficient scavenging of mtROS during hypoxia is complemented by increased production of ROS at reperfusion [87]. Mitochondrial loss of cyt c is considered to potentate ROS production at reperfusion because (a) cyt c is a catalytic scavenger for mitochondrial O_2_^•−^, and (b) loss of cyt c results in highly reduced state of respiratory complexes I, II, and III, thus, favoring electron release to molecular oxygen and O_2_^•−^ production [88, 89]. These observations suggest that mitochondrial inhibition of respiration and ATP synthesis resulting from hypoxia, coupled with an increase in O_2_^•−^ formation and ROS-induced injurious effects during reperfusion, potentially contribute to the contractile dysfunction and cell death in Chagasic hearts, to be confirmed in future studies. 

## 7. Summary

Sustained ROS generation of inflammatory and mitochondrial origin, coupled with an inadequate antioxidant response, result in the inefficient scavenging of ROS in the heart, and lead to long-term oxidative stress, and subsequently, to oxidative damage of the cardiac cellular components during chagasic disease. The alterations in biomarkers of oxidant and antioxidant status and in respiratory complex activities in the heart and blood/plasma of infected host appear to have same pathologic tendencies, which led to the suggestion that peripheral blood would be a useful tissue for investigating the pathologic importance of impaired mitochondrial function and oxidant/antioxidant status in chagasic disease development. Further studies should examine the pathological relevance of oxidative stress in clinical severity of chronic heart disease in Chagasic patients.

## Abbreviations

CI: NADH ubiquinone oxidoreductase
CII: Succinate decylubiquinone 2, 6 dichlorophenolindophenolreductase
CIII: Ubiquinol cytochrome c oxidoreductase
CIV: Cytochrome c oxidase
cyt c: Cytochrome c
GSH: Glutathione
GPx: Glutathione peroxidase
HNE: 4-hydroxynonenal
MDA: Malonyldialdehyde
MPO: Myeloperoxidase
NADH: Nicotinamide adenine dinucleotide (reduced form)
NOS: Nitric oxide synthase
PBN: Phenyl-*N*-*tert*-butylnitrone
ROS: Reactive oxygen species
SOD: Superoxide dismutase
*T. cruzi*: *Trypanosoma cruzi.*
